# Getting the Message Across: Outcomes and Risk Profiles by Awareness Levels of the “*Measure-Up*” Obesity Prevention Campaign in Australia

**DOI:** 10.1371/journal.pone.0121387

**Published:** 2015-04-06

**Authors:** Anne C. Grunseit, Blythe J. O’Hara, Josephine Y. Chau, Megan Briggs, Adrian E. Bauman

**Affiliations:** 1 Prevention Research Collaboration, School of Public Health, University of Sydney, Camperdown, New South Wales, Australia; 2 Health Marketing Unit, People, Capability and Communication Division, Department of Health, Canberra, Australian Capital Territory, Australia; University of Cambridge, UNITED KINGDOM

## Abstract

**Background:**

Obesity campaign evaluations have used campaign awareness to assess impact, yet have not compared unprompted campaign recallers, with prompted recallers and those with no campaign recall. Using data from an Australian mass-media obesity prevention campaign linking waist circumference and chronic disease we examined whether those with different degrees of campaign recall are distinct groups demographically and for subsequent campaign effects.

**Methods:**

A national cross-sectional telephone survey of randomly selected adults aged 18 to 65 years was conducted post- campaign (*n* = 2812) covering campaign recall, self-reported diet and physical activity (PA) and waist-measuring knowledge, behaviours and intentions to make lifestyle changes. Respondents were divided into three groups indicating campaign recall: Unprompted Recallers (n=1154); Prompted Recallers (n=1284); and No Recallers (n=374) and compared on demographic, knowledge, and behavioural risk factors for obesity/chronic disease.

**Results:**

Unprompted Recallers were more likely to speak English at home (p<.001), be in the primary campaign target group (25-45 years with children) (p<0.001) than the other two groups and to be university educated and female than the Prompted Recall group only (p=0.001). Unprompted Recallers had better knowledge about recommended waist circumference (p<.001), fruit (p=0.004), vegetable (p<0.001) and PA guidelines (p<0.001) than both the other groups. The No Recall group was less likely than the other two to be overweight/obese (46% vs 55%, p=0.020 and 54%, p=0.037), comparable on meeting fruit consumption and PA guidelines but more likely to meet vegetable intake recommendations (than Unprompted Recallers only).

**Conclusions:**

Unprompted recallers were more knowledgeable about campaign messages; behaviour change and intentions to change were stronger for the two recall groups compared with the No Recall group but not different between them. The current analysis revealed subtle differences in campaign exposure and/or attendance by different demographic subgroups that would not be apparent in a simple aware/unaware dichotomy.

## Introduction

The prevalence of overweight and obesity in Australia, after rising steadily since 1990 [1] remains at a worryingly high level [2] placing a large proportion of the Australian adult population at risk of chronic disease and ill health. In 2011–2012, 70.3% of Australian men and 56.2% of women were classified as overweight or obese.[3]

Modifiable lifestyle-related risk factors for obesity such as PA and diet are the target of many interventions conducted at the individual, institutional and community level.[4–6] Social marketing campaigns incorporating mass media have also targeted lifestyle behaviours in order to tackle the obesity problem and its health consequences [7]. Evidence suggests that mass media campaigns can have a positive, if modest, effect on health knowledge, beliefs, intentions and behaviours in health,[8] although studies evaluating mass media obesity prevention campaigns appear infrequently in the scientific literature.[9, 10]

*Measure-Up*, an obesity prevention campaign run in Australia between 2008 and 2010, focused on waist circumference as an indicator of an unhealthy lifestyle and chronic disease risk (www.measureup.gov.au). Formative qualitative research indicated broad appeal of information about a “healthy waist circumference” as a compelling, credible and easy to understand goal and the salience of the consequences for the *Measure-Up* characters regarding their progressive weight gain over time.[11] An evaluation of *Measure-Up* in one Australian state (New South Wales (NSW)) demonstrated it communicated new information about waist circumference and risk of chronic disease but population-level lifestyle-related behaviours were little changed following the campaign.[12]

“Campaign awareness” is the first step in the change process induced by social marketing and mass media campaigns and can therefore act as a proximal indicator of impact.[7, 10] Further, whilst attributing change in health behaviours to a single influence is problematic given their multi-determined nature, on average, if a campaign is effective, those aware of a campaign should report more short-term change in the desired direction compared with those who are not (the need to undertake change notwithstanding). Therefore, incorporating campaign awareness into impact evaluation analyses can indicate whether subsequent population-level change (or lack of it) could be due to campaign penetration (low or high reach) or the campaign content itself. That is, if behavioural outcomes remain unchanged post-campaign, in the context of low campaign awareness this could indicate poor targeting; in the context of high campaign awareness, absence of behaviour change could indicate ineffective content.

Campaign awareness in obesity campaigns has been estimated at around 60% (at first wave for continuing campaigns).[9, 13–15] Wammes et al (2007) showed that unprompted recall increased over the three years of their evolving weight gain prevention campaign (from baseline <40% to 72% at wave 6). This was less than *Measure-Up* which achieved 89% prompted recall [12] for wave 2. However, different campaigns have reported differing recall measures. For example, Van der Feen de Lille et al (“Fat Watch” campaign, 1998),[16] Van Wechem (“Fat Watch”, 1998)[13] and Croker et al, 2012 (“Change4Life” campaign)[17] reported recall as “recognition of the campaign name or tagline”, whereas Wammes et al (2007)[14] and Verheijden et al (2012)[18] for the “Don’t Get Fat” campaign, Morley et al (2009) (“Piece of String”),[9] King et al (2013) (“*Measure-Up*”)[12] and Wardle et al (“Fighting Fat Fighting Fit”, 2001)[15] reported both “unprompted” and “prompted” recall of campaign content. James et al (2011)[19] reported outcomes for those “aware of the Draw the Line campaign” (p e24) but did not describe prevalence of campaign awareness or how awareness was determined (prompted or unprompted).

Three obesity-prevention-mass-media campaigns have analysed the knowledge, perceptions and/or behaviours by campaign recall by combining those who recognised and those who recalled the campaigns and comparing them with those who were not exposed.[9, 14, 18] Wardle et al (2001)[15] presented demographic and weight data separately for those who recognised the campaign and those who recalled four campaign messages but did not formally compare them. Thus studies reporting the impact of mass media obesity campaigns vary in their measures of campaign recall, making cross-campaign comparison problematic.

To date there have been no comparisons of those who recalled an obesity campaign unprompted, compared with those recalling after prompting and those with no campaign recall. Using data from the *Measure-Up* campaign, the current analysis examines whether prompted and unprompted recallers are in fact distinct groups demographically and in terms of subsequent campaign effects.[20, 21] Understanding the associations between campaign recall and subsequent outcomes enhances evaluation of obesity campaigns by signalling whether these two types of recall identify different levels of message uptake.[22, 23] Further, we tested whether risk profile differs between levels of obesity campaign recall, addressing whether those reached by the campaign were those most in need.

Specifically, the following analysis investigates: 1) the demographic profile of the groups with different levels of recall of the Measure-Up campaign; 2) whether there is a dose-response effect of campaign recall on knowledge, attitude and behaviour uptake, 3) whether those who did not report recalling the campaign had a higher risk profile than those who recall or recognise the campaign.

## Method

### *Measure-Up* campaign

*Measure-Up* was a national Australian mass media campaign targeting obesity prevention, and is described in detail elsewhere.[12] Campaign advertising ran from late October 2008 to April 2009 and utilised paid television and radio messages, magazines, online and out of home media. It promoted waist circumference as an objective indicator for risk of chronic disease and as an impetus to personalise the call to action. Motivation to improve nutrition and PA levels was canvassed as a strategy to reduce waist circumference and improve health. Public Health services and key non-Government organisations were funded to undertake local activities to support the campaign, distribute campaign materials, and support public relations in local events.

The primary target audience was 25–49 year old adults with children, with a secondary target audience of those aged 45–65 years. The first media wave in 2008 launched the 60- and 30-second television commercials across Australia at 600 Target Audience Rating Points (TARPS) over four weeks (as a measure of the volume of weekly television advertising scheduled to reach the target audience), followed by shorter television messages and other media (including radio messages, magazines, online and out of home media). Based on the media purchased, approximately 75–80% of the target audience would be likely to have seen the TV commercial at least once; approximately 65% were expected to see the magazine advertisement and 70–75% were estimated to have heard radio advertisements. Wave 2 (March to April 2009) had 150 TARPS in the first week and 100 TARPS in the subsequent three weeks. Estimated TV reach was 72–77% of the target population, estimated magazine reach was 77% and estimated radio audience was 70–75%.[24]

### Evaluation study design

The campaign was evaluated via cross-sectional telephone surveys before and after the campaign of randomly selected adults aged 18 to 65 years. Baseline data were collected in October 2008, and follow up in April 2009. A report describing population level impact on recall, intentions and behaviour change in only one Australian state (NSW) has been published. [12] In this campaign recall study, national-level post-campaign data following wave 2 are used (n = 2812).

### Study population and sampling

This analysis is based on an Australian sample of adults with landline telephones. Households were contacted using random digit dialling (RDD) and sampling was based on population representative quotas for age, gender and state and for metropolitan and regional areas with stratification so that smaller locations had a robust sample size. Up to five call attempts were made to each generated number, and where there was more than one eligible respondent in the household, the “next birthday” technique was used to select a participant.[25]

### Measures

#### Recall of *Measure-Up* campaign

Unprompted recall of the *Measure-Up* campaign was assessed by asking if the respondent had seen, heard or read: “*any advertising campaigns about lifestyle*, *being overweight and chronic disease*” in the last month. Participants responding in the affirmative were asked to describe the advertising. The open-ended responses were systematically coded separately by three researchers (BOH, LK, AZ) and then cross-validated as identifiably from the *Measure-Up* campaign. The three reviewers independently coded the open-ended responses in line with a pre-determined coding framework. Any discrepancies in coding were discussed and an agreed resultant coding was used. The level of disagreement regarding the coding between the researchers was minimal. Responses considered relevant were references to the creative execution of the advertisement (e.g. “man walking along measuring tape”) and/or messages about waist size and risk of chronic disease. Prompted recall was assessed through asking respondents if they recalled seeing or hearing specific Measure Up advertisements used in all media channels.

The *Measure-Up* campaign recall groups at follow-up were formed as follows:

Unprompted Recall: 1) Responded to a screening question that they had seen an advertisement about lifestyle, being overweight, and chronic disease and, 2) described, unprompted, the main messages or images from the campaign.
Prompted Recall: 1) Either said that they had not seen an advertisement about lifestyle and being overweight and chronic disease OR said they had seen such advertising but did not describe *Measure-Up*, and 2) reported recognising the advertisement when prompted.
No Recall: Did not recall the *Measure-Up* campaign advertisements prompted or unprompted.

#### Knowledge

Knowledge of the health information conveyed in the *Measure-Up* campaign (www.measureup.gov.au) and supported by research [26] and current guidelines [27–29] was assessed by open ended questions recoded to dichotomous correct/incorrect variables as follows: waist measurement associated with increased risk of chronic disease (94cm for men and 88cm for women); serves of vegetables (five serves/day), fruit (two serves/day) and moderate/vigorous activity recommendations for health (150 minutes/week); and the chronic diseases caused by an unhealthy lifestyle (at least one of type 2 diabetes, heart disease and/or cancer coded as correct). Definitions of a serve or fruit and vegetables were provided to all participants and answers were coded as correct only if they were exactly correct; that is answers nominating an amount of fruit/vegetables or PA higher (or lower) than national recommended guidelines were coded as incorrect. Thresholds for at-risk waist measurement for men and women although asked of all respondents, were gender-specific for the current analysis.

#### Self-reported past behaviours and future intentions

Fruit and vegetable consumption were coded as responses meeting current Australian Dietary Guidelines of two serves of fruit and five serves of vegetables per day. Daily fast and/or snack food consumption response categories ranged from “don’t eat fast food”, less than one item, one, two, three, more than three items (specify) recoded to consuming ‘one or more items per day’ versus ‘less than one item’. All food consumption questions included a definition of what constituted a serve of fruit, vegetables and a fast food item. Active Australia questions were used to assess PA using established analytic protocols.[30] Respondents were classified ‘sufficiently physical active’ if they reported at least 150 minutes of total weekly PA.[31]

Respondents were also asked whether they had tried to increase their PA and consumption of fruit and vegetables, decrease the amount of fast and snack food, measured their waist in the last six months (and what that measurement was) and whether they intended to do so in the next six months.

#### Risk profile and risk perception

Self-reported height and weight were asked, and BMI was calculated according to standard definitions.[32] Satisfaction with current waist circumference on a five-point scale from ‘very satisfied’ to ‘very dissatisfied’ and self-rating of general health were also asked.[33] Three items examined perceived personal susceptibility to chronic disease as follows: 1) I have a high chance of developing a chronic disease, 2) I am concerned that I will develop a chronic disease and 3) My lifestyle is increasing my risk of chronic disease. The questions were designed to elicit respondents’ perception of their own risk of developing a chronic disease, a key target for campaign message development identified in formative research.[34] Responses were on five-point Likert scales, rated from ‘strongly agree’ to ‘strongly disagree’. For analysis ‘strongly agree’ and ‘ agree’ were collapsed together as were ‘strongly disagree’, ‘ disagree’, ‘don't know’ and ‘neither agree nor disagree’ to form binary indicators for each item reflecting a ‘concerned’ versus a ‘lack of concern’ dichotomy.

### Statistical analysis

Bivariate associations between campaign recall group (Prompted Recall, Unprompted Recall, No Recall) and demographic characteristics were tested using chi-square statistics. Contrasts between groups over all dichotomous demographic, knowledge, behaviour and intention variables were conducted using generalised linear models using a log link and binomial distribution [35] with level of recall entered as a categorical variable and the Unprompted Recallers as the reference group. Where models did not converge, Poisson models with robust variance estimators were used.[36] To test for a dose-response effect for campaign recall, models were re-run with campaign recall group entered as an ordinal variable (in ascending order from least to most recall) for campaign-related knowledge, behaviours and intentions. Analyses examining the risk profile and perceptions of the No Recall group compared with the other two groups used the same techniques, with No Recall designated as the reference category. Regression results are described as prevalence ratios. All analyses were carried out using Stata version 11.2,[37] and given that the analysis was essentially exploratory, a significance threshold of 5% was set. Data were weighted to the 2006 Australian Bureau of Statistics (ABS) population distributions by gender, age and location (state/metropolitan vs non- metropolitan) in Australia.[38]

### Ethics approval and consent

This secondary analysis of the *Measure-Up* evaluation data collected by the Australian Federal Department of Health was approved by the Sydney University Human Research Ethics Committee. Participants provided verbal rather than written consent to the original survey as the study was conducted over the telephone with recruitment through random-digit dialing. The current study is a secondary analysis of the data collected and did not seek further consent as the purpose of re-analysis was no different from the purpose stated when originally seeking consent. This approach to consent was approved by the Sydney University Human Research Ethics Committee.

## Results

The response rate, as a proportion of the 7769 eligible households able to be contacted (those completed plus those who refused), was 36% (n = 2812).[39] No information was available on the eligibility or demographic make-up of those who refused or household who could not be contacted. The demographic characteristics of the post-campaign sample are shown in Table 1. Half were male, over one-third reported a university education, two-thirds were in paid employment and half of the sample self-reported as overweight or obese (53%). Consistent with the quota sampling approach outlined above, the sample compared well to population distributions for sex (within 0.1%) and age (within 1.2%) [38].

**Table 1:** Unweighted demographic characteristics and (weighted) distribution of BMI in post-campaign sample.

DEMOGRAPHIC CHARACTERISTIC	n	%
**All**	2812	100
**Gender**		
Female	1,403	49.9
Male	1,409	50.1
**Age (in years)^1^**		
18–24	418	14.9
25–34	614	21.8
35–44	666	23.7
45–49	294	10.5
50–54	323	11.5
55–65	497	17.7
**Highest level of education**		
Less than 12 years schooling	500	18.1
12 years school/apprenticeship/certificate/diploma	1,269	45.9
University degree or higher	994	36.0
**Employment Status**		
Not in paid employment	815	29.1
In paid employment	1,989	70.9
**Annual household income^2^**		
<$50k	598	21.3
$50k—< $100k	1,006	35.8
$100k +	775	27.6
Don’t know/refused	433	15.4
**Language spoken at home**		
English	2,620	93.2
Other than English	190	6.8
**Location**		
Metropolitan	1324	47.1
Non-metropolitan	1488	52.9
**BMI^3^**		
Underweight/average	1,176	47.1
Overweight	847	33.2
Obese	530	19.7

Notes

^1^ Secondary campaign target group aged 45–65 years.

^2^ Income was collected as: <$AUD30,000; $AUD30,000–49,999; $AUD50,000–69,999; $AUD70,000–99,999; $AUD100,000+. The categories <$50k, $50k—< $100k, $100k+ approximate to: low income (below the 40^th^ percentile), middle income (between 40^th^ and 70^th^ percentile) and high income (70^th^ percentile and above) gross annual income for the Australian population.[40]

^3^ Missing 9.2% (n = 259) for self-reported height/weight, weighted percentages

Without prompting, the *Measure-Up* campaign was recalled by approximately 41% of respondents (n = 1154) (Unprompted Recall), recalled after prompting by a further 46% (n = 1284) (Prompted Recall) while 13% (n = 374) reported neither recalling nor recognising the campaign (No Recall). Table 2 shows the demographic profile for each of the campaign recall groups.

**Table 2:** Demographic profile for each level of *Measure-Up* campaign awareness (n = 2812).

DEMOGRAPHIC CHARACTERISTIC	All†	No Recall†	Prompted recallers†	Unprompted recallers† (reference)	Chi-square
	n = 2812	n = 374	n = 1284	n = 1154	p
**Gender—% Male**	49.6%	49.5%	53.4%**	45.0%	0.008
**Age—% aged 45–65yrs^1^**	40.6%	47.9%**	44.2%**	32.5%	<.001
**Highest education—% university**	36.2%	37.0%	31.2%**	41.7%	<.001
**Language spoken at home—% not English**	6.8%	10.5%**	7.3%**	4.2%	<.001
**Employment—% employed**	69.5%	68.1%	68.8%	70.9%	0.579
**Household income—% >$100k**	30.5%	27.4%	29.9%	32.7%	0.271
**Parental status—% lives with own children**	38.9%	26.6%**	36.5%**	48.2%	<.001
**Primary target group^2^**	34.1%	23.3%**	32.0%**	42.2%	<.001
**Location—% capital city**	60.4%	72.9%**	57.3%	57.6%	<.001

† Weighted percentages

* Category significantly different from Unprompted Recallers at. 05,

** at. 01 in regression analysis

^1^ Secondary campaign target group

^2^ Primary target group are aged 25–49 with children younger than 17 years

There were overall significant differences among the campaign recall groups for all demographic characteristics with the exception of employment status and income (Table 2). Contrasts showed that specifically the Unprompted Recall group were more likely to be university educated and female than the Prompted Recall group but not the No Recall group, and were less likely to be located in an metropolitan area than the No Recall group although not the Prompted Recallers. Unprompted Recallers were more likely than the other two groups to be living with their children, speak English at home, to be in the primary target group (25–45 year old group with children), but less likely to belong to the secondary target group (45–65 year-old group). Employment and income did not vary significantly by recall.

Table 3 shows the knowledge, past behaviour and future intentions regarding diet and PA patterns across the three campaign recall groups, with a test for trend across level of recall. The prevalence ratio (PR) shows the percentage increase in prevalence for moving from the No Recall group to the Prompted Recall group to the Unprompted Recall Group. In general the Unprompted Recallers had better knowledge than both the No Recall group and those that only recognised the campaign after prompting and demonstrated a dose-response effect of between 5% and 73%. For example, the prevalence of correct responses for recommended daily intake of vegetables was on average 51% higher for each increment in campaign recall.

**Table 3:** *Measure-Up* campaign-related knowledge, behaviours, and intentions for each campaign recall group, with tests for trend.

CAMPAIGN OUTCOME	No Recall	Prompted Recallers	Unprompted Recallers (reference)	Trend	p trend
	n = 374	n = 1284	n = 1154	PR (95%CI)	
**Knowledge**					
Risky waist circumference (% correct)	8.8%**	13.2%**	24.3%	1.73 (1.46–2.05)	<.001
Vegetable guidelines (% correct)	20.7%**	29.1%**	45.2%	1.51 (1.36–1.67)	<.001
Fruit guidelines (% correct)	37.8%**	40.9%**	47.9%	1.14 (1.05–1.23)	0.001
Physical activity guidelines (% correct)	52.0%**	54.7%**	66.1%	1.15 (1.08–1.22)	<.001
Misperception of own weight	31.4%	29.6%	27.6%	0.94 (0.84–1.04)	0.230
Mentioned diabetes/cancer/CHD	85.7%**	90.2%**	95.0%	1.05 (1.03–1.08)	<.001
**Past behaviours**					
Measured waist in last 6 months	30.8%	38.0%	37.4%	1.08 (0.99–1.17)	0.092
Tried to increase vegetables last 6 months	29.1%**	36.2%	38.2%	1.12 (1.03–1.23)	0.009
Tried to increase fruit last 6 months	29.7%	30.6%	31.8%	1.04 (0.94–1.14)	0.496
Tried to decrease fast food last 6 months	35.8%**	46.7%**	53.9%	1.2 (1.12–1.29)	<.001
Tried to increase physical activity last 6 months	44.2%**	50.5%*	55.8%	1.12 (1.05–1.19)	0.001
**Future intentions**					
Increase vegetables in next 6 months	33.8%**	39.3%	43.8%	1.13 (1.04–1.23)	0.003
Increase fruit in next 6 months	27.5%	33.3%*	28.2%	0.98 (0.90–1.08)	0.751
Decrease fast food in the next 6 months	31.5%**	41.6%	42.2%	1.12 (1.04–1.21)	0.005
Increase physical activity in next 6 months	55.4%*	61.8%	63.7%	1.06 (1.01–1.12)	0.021
Measure waist in next 6 months	25.4%**	34.7%	35.7%	1.14 (1.05–1.25)	0.003

* Category significantly different from Unprompted Recallers at. 05,

** at. 01

With respect to behaviour change, proportionately fewer respondents in the No Recall and Prompted Recall groups reported trying to improve their fast food intake or PA in the last six months compared than Unprompted Recallers. The No Recall group was also significantly less likely to report attempting to improve their vegetable intake in the last six months compared to the Unprompted Recallers, but Prompted Recallers and Unprompted Recallers were comparable across the other behaviours (Table 4). Supplementary analyses comparing the two recall groups only on behaviours respondents reported changing “as a result of seeing this campaign” (data not shown) confirm this with no significant differences for trying to increase fruit or vegetable consumption, and PA and one small but significant difference for waist measuring (Unprompted Recall: 18.5% vs Prompted Recall: 13.9%, p = .013). Intentions in the next six months showed a pattern where Unprompted Recallers were more likely to intend to change their behaviours towards a healthier pattern (e.g., higher vegetable intake) than No Recall but not Prompted Recall, with the exception that a higher proportion of the Prompted Recallers than Unprompted Recallers intended to increase their fruit intake (33% vs 28% respectively, p = .004). There were significant trend effects for trying to increase waist measuring, decreasing fast food, increasing vegetables and PA in the next six months with between 12–14 percent more respondents reporting intending to improve per increment in recall of the *Measure-Up* campaign. However, the lack of significant differences for the contrasts and small effect sizes for Unprompted versus Prompted Recallers suggest that the significant results for trend were likely due to the No Recall group’s result rather than a true dose-response effect.

**Table 4:** Risk profile and risk perceptions for each level of *Measure-Up* campaign awareness.

CHARACTERISTIC	No Recall (reference)	Prompted Recallers	Unprompted Recallers	Chi-square
	n = 374	n = 1284	n = 1154	p
BMI category overweight/obese	46.1%	54.9%*	54.1%*	0.030
Eats ≥5 serves vegetables/day	12.4%	10.3%	7.6%*	0.037
Eats ≥2 serves fruit/day	56.0%	55.9%	63.0%	0.017
Eats fast food ≥1 times/day	36.9%	44.2%*	44.4%*	0.049
Physical activity ≥150 mins/week	70.7%	71.9%	74.8%	0.323
Self-perception as overweight	30.6%	36.5%	40.5%**	0.010
Rate general health poor/fair	20.4%	18.8%	18.7%	0.796
Dissatisfied with waist circumference	33.6%	33.0%	38.9%	0.057
I have a high chance of developing a chronic disease	36.0%	32.7%	32.8%	0.533
I am concerned that I will develop a chronic disease	35.2%	38.9%	42.9%*	0.059
My lifestyle is increasing my risk of chronic disease	32.8%	34.3%	37.2%	0.323

* Category significantly different from No Recall at. 05,

** at. 01

To examine whether those in the No Recall group are, and/or perceive themselves to be, at greater or lesser risk of obesity-related chronic disease than those who recalled (prompted or unprompted) the *Measure-Up* campaign, the risk profile and perceptions of risk for the three campaign recall groups were analysed (Table 4). The No Recall group had a significantly lower prevalence of being overweight/obese (46%) compared with Prompted Recallers (55%) and Unprompted Recallers (54%). A lower proportion of the No Recall group (37%) were eating fast food more than once a week compared with the other two categories (both 44%). Significantly more Unprompted Recallers than No Recallers reported being concerned that they would develop a chronic disease and perceived they were overweight (43% vs. 35%, respectively), despite comparability on perceived chance of developing a chronic disease (Table 4). The No Recall group reported consuming the recommended amount of vegetables than Unprompted Recallers, but was not different behaviourally than the other two groups on fruit consumption or meeting PA guidelines.

## Discussion

Confirming earlier research, the *Measure-Up* campaign reached approximately half of the Australian adult population with campaign relevant messages with this innovative approach to frame obesity-related chronic disease risk.[12] Both prompted and unprompted campaign recall were very high (around 87% combined). Analysis by level of recall showed different associations with demographic characteristics, risk profile and campaign-related knowledge, behaviour and intentions by degree of campaign recall. Further, the risk profile for chronic disease of those respondents unaware of the campaign was no poorer, and in some respects more favourable, than those aware of the campaign. From a methodological perspective, the analyses suggest combining prompted and unprompted recallers may mask informative differences between these groups irrespective of the sources of those differences, be they pre-existing receptivity to health messages, campaign exposure and/or message absorption.

Demographic heterogeneity may be found even within those designated as ‘aware’ of the campaign. The Unprompted Recall group had the highest proportion of the primary target group (adults aged 25–49 years with children), but the lowest proportion of the secondary target group (45–65years). That the *Measure-Up* campaign reached and was recalled by its target audience confirms the value of campaign investment resulting in high media message exposure.[8, 41] Recall rates were similar by gender, unusual for health campaigns as women are usually more likely to attend to health messages.[42, 43] In the case of *Measure-Up*, this finding may stem from greater salience for men of the male-modelled *Measure-up* advertisement. (http://www.measureup.gov.au/internet/abhi/publishing.nsf/Content/television). Further analyses found that men were more likely to recall only after prompting, whereas women were more likely to recall unprompted, once again suggesting closer attention to health campaign messages by women. Unprompted Recallers were more likely to have had a university education than Prompted Recallers but were comparable to those not recalling the campaign. Proportionately fewer people from non-English speaking backgrounds were among the Unprompted Recallers, perhaps reflecting the role of language proficiency in health literacy[44], lower identification with the (Caucasian) message role-models, and/or lower exposure to mainstream Australian television.[34, 45]

A key target group for health campaigns are people in non-metropolitan areas as they can less easily access health services.[46] *Measure-Up* reached a disproportionate number of residents from non-metropolitan locations (equal for prompted and unprompted recall) underscoring the important role of television advertising in social marketing in reaching rural populations. Indeed the free telephone lifestyle coaching service (“Get Healthy Service” (GHS)) advertised in tandem with *Measure-Up* in and GHS specific advertising for NSW showed the reach to non-metropolitan communities was reflected in uptake of obesity prevention support, a central step towards behaviour change.[47] Thus the current analysis revealed subtle differences in the exposure and/or attendance by different demographic subgroups that would not be apparent in a simple aware/unaware dichotomy.

The significance of differential recall among demographic subpopulations is found in the dose-response relationships for knowledge regarding the key messages from *Measure-Up*. Better campaign recall was consistently associated with correct responses for fruit, vegetable and PA guidelines and understanding the link between lifestyle and chronic disease. However, the corresponding self-reported behaviours followed a slightly different pattern, with Unprompted Recallers more likely to try to change their behaviours towards healthier practices compared to No Recallers, confirming findings from evaluations of other weight related campaigns.[9, 14, 18) For example, in the Dutch national “Don’t Get Fat” campaign’s second phase, low SES respondents reporting exposure to the campaign showed an increase in their attention to food choices compared to those reporting no campaign exposure; in the longer term, BMI increases were less likely among respondents from non-Dutch backgrounds with some exposure to the campaign relative those reporting no message exposure.[18]

Despite greater recall, with only a few exceptions, Unprompted Recallers and Prompted Recallers were comparable on past attempts and future intentions to change their health behaviours, a result further confirmed when behaviour change was narrowed specifically to that which the respondents said was “as a result of seeing this campaign”. Hence, while reporting exposure to the advertisement was correlated with reported or intended behaviour change, despite their greater knowledge, the Unprompted Recall group did not report greater efforts to change than the Prompted Recallers.

From a health promotion perspective, the lower fruit consumption reported by the Prompted Recall group was matched by an intention to increase fruit intake in the next six months. Of greater concern was the small proportion across all groups that met vegetable consumption guidelines, yet less than half (≈40%) reported intending to increase their vegetable intake. Adequate vegetable intake remains one of the more difficult lifestyle factors to change[48] despite knowledge of recommended intake and positive intentions. Thus the priming steps generated by mass media campaigns such as *Measure-Up* require further translation to behaviour change with community and environmental supportive programs and policies, or access to community-wide programs (e.g. GHS);[47] unfortunately these data did not include whether the respondent contacted a support service such as GHS.

The No Recall group did not appear to be at any greater risk of chronic disease than their campaign-aware counterparts. Indeed, for BMI, vegetable (vs Unprompted Recall only) and fast food consumption they fare significantly better than the two recall groups and the population in general (at least for BMI).[2] Thus, it would appear that those unaware of the *Measure-Up* campaign were not the intended target group of the campaign, and may have not attended to the campaign (if they had seen it) because of a perceived and actual lower than average prevalence of overweight/obesity (although still 46%). Conversely, the primary target group as identified by pre-campaign formative research, who constitute those in the Australian population most at risk of lifestyle related chronic disease, was better represented among those who could recalled the campaign than those who did not; 87% of this group recalled the campaign (unprompted + prompted, data not shown).

### Strengths and limitations

To our knowledge, this study is the first to analyse a national obesity campaign that compares outcomes for prompted and unprompted recall as well as those unaware of a campaign. Other strengths of the study were the large, nationally representative (for age/sex/state) sample although the rate of obesity/overweight was marginally lower than reported in other general population surveys and the current sample had a higher proportion who had a degree than the general Australian population. [49] Hence the sample reported on here may have better health literacy and/or interest in health messages than the general population.[50] However despite this, the relationship between campaign recall and outcomes are likely to be generalizable. [51] This was one of the few evaluations of obesity campaigns reported in the literature. *Measure-Up* represents a significant investment in social marketing for the Australian Government and therefore an important target for evaluation. One limitation is that the cross-sectional design which, whilst good for avoiding the effects of sensitisation to the campaign[52] does not allow for a rigorous analysis of the determinants of individual-level change. For example, the better knowledge of health recommendations of the Unprompted Recall group may predate *Measure-Up* and therefore this groups’ prior interest in health issues led to their better recall of the campaign. Moreover, the extent to which a person may need to adjust their lifestyle or BMI cannot be disentangled from behavioural outcomes as the causal sequence cannot be established in a cross-sectional design, only correlations. However, the clear dose-response effects of recall observed suggest that the direction of effect was that *Measure-Up* improved knowledge. Further, the aim of the analysis was not to examine the correlates of intended or reported behaviour change but rather characterise the different recall groups and examine whether a “no recall” group was any more at risk than those reporting recalling the campaign. Future longitudinal designs could build on this research and examine interactions between campaign exposure/recall, risk profile and subsequent behaviour change. Finally the use of RDD sampling frame will have resulted in systematic exclusion of mobile phone-only households. Although there is some evidence that samples of landline households may underestimate prevalence of some unhealthy behaviours,[53] this should not have unduly biased the results in this analysis as the proportion of mobile-phone only households at the time of the survey in Australia was relatively small.[54]

## Conclusion

Mass media campaigns are an important first step in communicating to the community about complex public health problems such as obesity. *Measure Up* was the initial step in a multi-year preventive approach to obesity in Australia. In this analysis, better recall of the *Measure-Up* campaign was associated with better knowledge, about campaign messages; behaviour change and intentions to change were stronger for the two recall groups compared with the No Recall group but not different between them. Moreover, the demographic profiles of the two recall groups suggest that campaign messages have differential penetration with exposure. From an analytical perspective, differentiating between prompted and unprompted recallers in impact analysis and profiling campaign reach may reveal useful insights into message uptake among different subgroups, although behaviour change did not necessarily flow from greater message uptake. Further, examining whether those unexposed or at least not recalling a campaign are at any greater risk than their exposed counterparts is a key element in evaluating the reach. While numbers may prevent some campaign evaluations from partitioning those recalling a campaign into prompted and unprompted, investigating demographic profiles and impacts in this fashion may afford greater insights into the relationships between exposure, recall, uptake of campaign messages and behavioural change.
